# Splenic metastasis of ovarian clear cell adenocarcinoma: A case report and review of the literature

**DOI:** 10.3892/etm.2014.1500

**Published:** 2014-01-24

**Authors:** MEILING LV, YUYAO LI, CHANGQIN LUO, PEIJUN LIU, JIN YANG

**Affiliations:** 1 Department of Clinical Oncology, The First Affiliated Hospital of Xi’an Jiaotong University College of Medicine, Xi’an, Shaanxi 710061, P.R. China; 2 Clinical and Translational Science Center, The First Affiliated Hospital of Xi’an Jiaotong University College of Medicine, Xi’an, Shaanxi 710061, P.R. China

**Keywords:** ovarian clear cell carcinoma, splenic metastasis, cytoreductive surgery, splenectomy

## Abstract

Splenic metastasis of ovarian cancer appears to be more common in serous cystadenocarcinomas. Splenic metastasis usually occurs postoperatively and simultaneously with dissemination to the greater omentum and pelvic cavity. Compared with other ovarian cancer subtypes, ovarian clear cell adenocarcinoma (OCCA) is rare, accounting for <5% of all ovarian malignancies. OCCA has a distinct histological type with poor prognosis and resistance to platinum-based chemotherapy. In the present study, a case of isolated splenic metastasis of OCCA was reported. A 53-year-old female presented with a mass in the left upper quadrant without any other clinical manifestations. Subsequent abdominal and pelvic computed tomography scans revealed multiple mixed cystic-solid lesions, potentially predicting ovarian malignancy. Pathological tests following ovarian cytoreductive surgery revealed primary OCCA with metastases to the spleen. The current study also reviewed recently published literature on splenic metastasis of ovarian carcinoma and demonstrated that the reported case was a rare case of isolated splenic metastasis of OCCA.

## Introduction

Ovarian cancer is a common gynecological malignant tumor that presents at a late clinical stage in >80% of the total patient population (1). It is associated with a 5-year survival rate of 35% in advanced ovarian cancer patients. The incidence of ovarian cancer ranks third while the mortality rate is the highest of all gynecological malignant tumors. The National Comprehensive Cancer Network 2011 guidelines revealed that epithelial ovarian carcinoma (accounting for 80% of all malignant ovarian tumors) is the leading cause of mortality among gynecological cancers in the USA (2). In total, <40% of patients with epithelial ovarian carcinoma are completely cured (3,4). The occurrence of ovarian clear cell carcinoma (OCCA) is relatively infrequent, accounting for 5% of all ovarian malignancies and 3.7–12.1% of all epithelial ovarian carcinomas (5–7). As reported previously, OCCA has a distinct histological type with poor prognosis and resistance to platinum-based chemotherapy (7,14). The majority of patients with advanced or recurrent OCCA have a low benefit-to-failure ratio from palliative chemotherapy (7).

According to clinical data, splenic tumors metastasized from ovarian cancer are uncommon, accounting for 2–4% of all malignant tumors of the spleen with an incidence of 0.6% in autopsy studies and ~1.1% in splenectomies (8). In the present study, a rare case of splenic metastasis of OCCA was reported and an extensive review of the literature on splenic metastases of ovarian carcinomas published in the past decade was performed. The aim was to improve the diagnosis and treatment of splenic metastasis of ovarian cancer.

## Case report

### Patient history

A 53-year-old female was admitted to the First Affiliated Hospital of Xi’an Jiaotong University, (Xi’an, China) after presenting with a mass in the left upper quadrant which had been there for three months. The patient did not present with any other clinical manifestations. The study was conducted in accordance with the Declaration of Helsinki and was performed with approval from the Ethics Committee of Xi’an Jiaotong University. Written informed consent was provided by the participant.

### Examination

**Figure 1:**
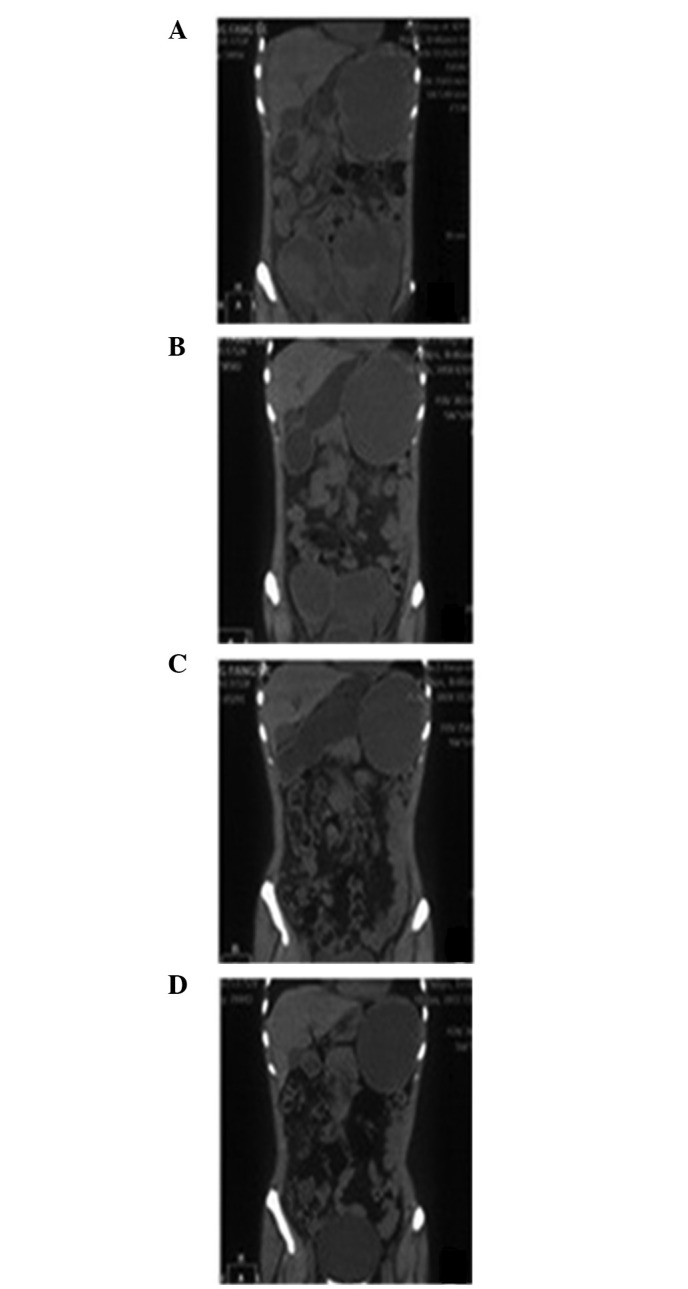
Primary pelvic and metastatic splenic tumors prior to and following chemotherapy. (A) Prior to chemotherapy, extensive lesions with vague boundaries were detected in the pelvic cavity and mixed cystic-solid masses were identified in the spleen. Following (B) two and (C) four cycles of chemotherapy, changes occurred in the lesions in the pelvic cavity and spleen. (D) Following six cycles of chemotherapy, the primary pelvic tumor was markedly degraded and the splenic mass gradually became cystic.

An abdominal B-ultrasound, performed prior to hospitalization, revealed multiple mixed cystic-solid lesions in the left upper quadrant and the lower abdominal region. A needle biopsy of the mass revealed hyperplasia of fibrous granulation tissue, infiltration of chronic inflammatory cells, focal shape necrosis, active growth and tumor-like hyperplasia in sections of the cells. The histological features prompted the consideration of a mesenchymal tissue tumor. Following admission, the patient had a cancer antigen (CA)-125 level of up to 4,712 U/ml (normal <35 U/ml). Subsequent abdominal and pelvic computed tomography (CT) scans revealed multiple mixed cystic-solid lesions and abdominal and pelvic cavity effusion, presumably due to an ovarian malignancy and multiple metastatic intraperitoneal tumors (Fig. 1, prior to chemotherapy). Pathological results of the needle biopsy on the pelvic mass revealed a level II papillary adenocarcinoma accompanied by necrosis.

### Treatment

**Figure 2:**
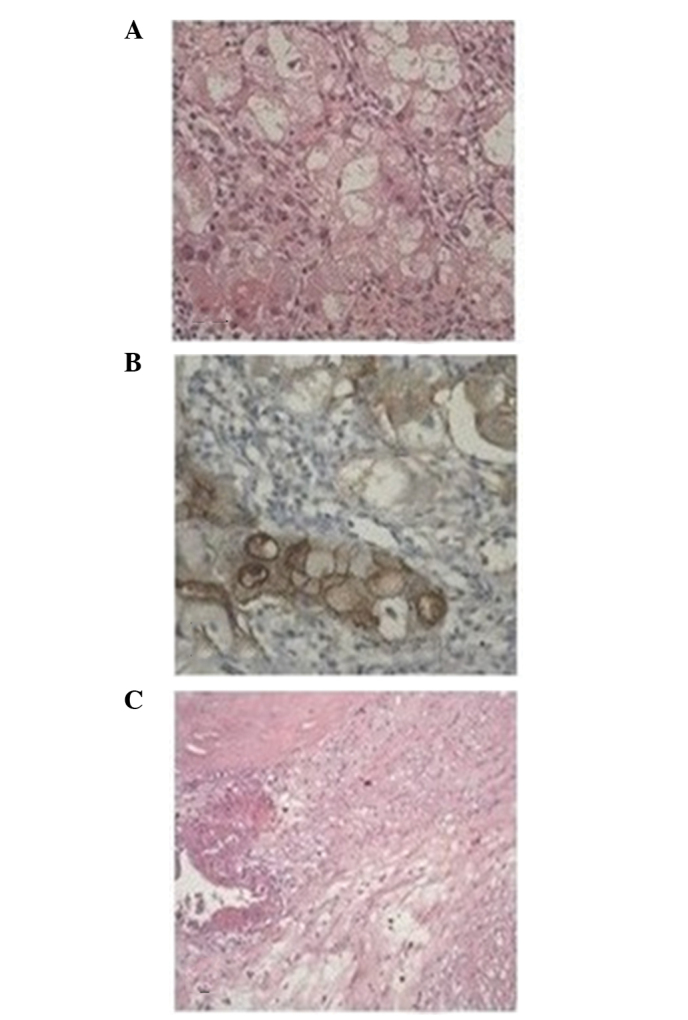
Postoperative pathological results of the lesions. (A) Ovarian pathology following cytoreductive surgery (H&E staining; magnification, ×400). (B) CA-125 expression in ovarian clear cell carcinoma (immunohistochemical staining; magnification, ×400). (C) Splenic pathology following splenectomy (H&E staining; magnification, ×200). H&E, hematoxylin and eosin; CA-125, cancer antigen-125.

The patient was diagnosed with ‘abdominal and pelvic cavity metastasis of ovarian carcinoma’ and administered six cycles of systemic chemotherapy (TP scheme). The patient received 100 mg docetaxel (day l) and 40 mg cisplatin (days 1–2) for 21 days in each cycle. During the course of therapy, the CA-125 level decreased from 4,712 U/ml to 39.42 U/ml. Follow-up CT reexamination demonstrated that the lesions had markedly diminished, thus, the therapeutic evaluation was partial remission (PR) (Fig 1). Subsequently the patient underwent ovarian cytoreductive surgery in addition to a splenectomy. The final pathology results revealed OCCA (oxyphil cell type) with massive necrosis and splenic metastasis of OCCA (oxyphil cell type) with massive necrosis, which were in accordance with the chemotherapeutic results. The immunohistochemical results were positive for CA-125, cytokeratin (CK)7, CK19 and epithelial membrane antigen, but negative for CK20 and CD68 (Fig. 2).

## Literature review

Case reports and literature reviews on splenic metastasis of ovarian carcinoma, which had been published in the past decade, were collected. Based on the search results, preliminary analysis of the clinical characteristics of splenic metastasis of ovarian carcinoma was conducted in 34 cases (Table I).

In total, 13 case reports were retrieved on splenic metastasis of ovarian cancer published in Western countries. Although each case had distinctive features, pathologically, the majority were serous papillary adenocarcinoma (9–16,18,20) with only one case of angiosarcoma (17) and one case of carcinosarcoma (19). No case reports regarding OCCA were found. Splenic metastasis occurred postoperatively with the exception of one case in which splenic metastasis occurred simultaneously with radical resectioning of ovarian cancer. Preliminary analysis was also conducted on 21 cases in ten case reports of splenic metastasis of ovarian cancer published in China over the past decade. The analytical results were largely consistent with the data from the case reports published in Western countries. Pathologically, these cases were serous papillary cystadenocarcinomas with two cases of mucinous papillary adenocarcinoma. Splenic metastasis primarily occurred years after surgery. Splenic metastasis was only detected in four cases when ovarian cancer was first diagnosed.

OCCA is infrequently reported in literature owing to its low incidence. Pathologically, the majority of cases in the reports on splenic metastasis of ovarian cancer belong to serous papillary cystadenocarcinoma and only individual cases are poorly differentiated transitional cell carcinomas. Based on the present analyses of the associated case reports and literature reviews, we hypothesize that splenic metastasis of ovarian carcinoma largely occurs postoperatively, following subsequent surgeries or years after radiotherapy and chemotherapy. Splenic metastasis is primarily accompanied by dissemination to the omentum majus and pelvic cavity. Isolated metastatic splenic lesions only occur in a few individual cases.

## Discussion

OCCA is a malignant tumor found primarily among elderly females. It was formally defined as a special type of ovarian cancer in 1973 by the World Health Organization (21–23). OCCA originates from the Müllerian duct and is closely associated with endometriosis (24). Compared with other adenocarcinomas, OCCA has unique biological features, resulting in poor prognosis and high rates of recurrence and metastasis (25). The main therapeutic strategies focus on surgery and chemotherapy, although OCCA is relatively resistant to conventional platinum-based chemotherapy (7,26). In the present study, a rare case of splenic metastasis of OCCA was reported. No experience or rules concerning the treatment and prognosis of OCCA were available to follow. Thus, the aim was to provide new ideas for the treatment and prognosis of this rare disease.

The case reported in this study is valuable due to the unusual pathological type of OCCA. Dissemination to the spleen and pelvic cavity were detected at the initial diagnosis. The case was once misdiagnosed by needle biopsy, indicating that needle biopsy alone may easily lead to misdiagnosis for special pathological types of ovarian cancers, including OCCA, with splenic metastasis.

Compared with easily affected organs, including the liver and lung, the spleen is rarely affected (27) with an incidence of ~0.6% in autopsy studies and 1.1% in splenectomies (8). Primary tumors with splenic metastases, systematically reported in Western countries, are mainly melanomas and lymphomas, followed by breast, lung and ovarian cancers (28). It is generally recognized that among the routes of metastasis, hematogenous metastasis ranks first, followed by lymphatic and implantation metastases. Reasons for the rare occurrence of splenic metastases are as follows. Firstly, the sharp angle made by the splenic artery makes it difficult for tumor emboli to enter the spleen. Secondly, the rhythmic contractile nature of the spleen squeezes out the tumor embolus and prevents the tumor lodging in the spleen. Thirdly, the absence of afferent lymphatics that bring metastatic tumors to the spleen. Finally, antitumor activity due to the high concentration of lymphoid tissue in the spleen (29). It has been hypothesized that metastatic tumors rarely grow in the spleen since the spleen is a pharmacological and immunological sanctuary. Once a metastatic splenic tumor grows, it may indicate that monoclonal slow-growth is occurring (30).

There is no unified treatment and prognosis for late-stage ovarian cancer with splenic metastasis due to the low incidence. Therefore, more clinical studies are required. Bristow *et al* (31) confirmed that the thoroughness of cytoreductive surgery is key to prognosis. Thus, a more thoroughly performed surgery is likely to lead to a better prognosis. Destruction of 10% of the cancer cells enhances the median survival rate by 5.5%. Patients in whom >75% of the cancer cells have been eliminated have a median survival time of 33.9 months, while patients in whom <75% of the cancer cells have been eliminated have a median survival time of 22.7 months (P<0.05). However, in more than two-thirds of patients with ovarian cancer, the lesions have already spread prior to undergoing primary surgery. Thus, in order to excise all metastatic lesions, cytoreductive surgery involves the pelvic and abdominal cavities, including a splenectomy, diaphragmatic surgery and partial hepatectomy. Splenectomies, based on the accurate evaluation of a patient’s condition, may improve the patient’s median survival time and quality of life. However, the decision to perform surgery should be made cautiously. If satisfactory removal surgery cannot be achieved, priority should be given to palliative surgery and chemotherapy.

In the case of the present study, following six cycles of TP chemotherapy, the CA-125 levels decreased gradually to within a normal range. Imaging evaluation revealed that the curative effect reached PR. In addition, the patient underwent cytoreductive surgery and a splenectomy and the surgical results were satisfactory. In the following 8 months of follow-up, the patient was in a good condition without any sign of recurrence and metastasis. The patient continues to be followed-up and further observation is required for the long-term survival.

In conclusion, splenic metastasis of ovarian cancer may be diagnosed by a combination of clinical history, imaging information and histopathology. For space-occupying lesions of the spleen, CT is capable of demonstrating intraparenchymal and infiltrative splenic metastasis in patients with ovarian cancer, even in the absence of increased CA-125 levels (32,33). While aspiration cytology is not recommended, complete cytoreduction in primary or subsequent surgeries is an ideal treatment for space-occupying lesions of the spleen; even for recurrent carcinomas. Cytoreductive surgery is capable of prolonging progression-free survival times and improving quality of life (34–37). Due to the immunological function of the spleen, the formation of a metastatic splenic carcinoma usually indicates an advanced stage of the disease that has poor prognosis. In such cases, a splenectomy followed by a comprehensive therapy is the preferred course of treatment. Therapeutic treatments may be more accurately determined based on the consideration of the primary lesions, the general condition of the patient and whether multiple metastases have occurred in other organs.

## Figures and Tables

**Table I:** Clinical characteristics of splenic metastasis of ovarian carcinoma in 34 cases published in the past decade.

Case	Age (years)	Pathology	Time of postoperative splenic metastasis (years)	Characteristics of splenic metastasis (ref.)
1	52	Serous	20	Solitary (10)
2	85	Serous	Simultaneity	Solitary (11)
3	38	Serous	3	Solitary (12)
4	29	Serous	1	Solitary (13)
5	61	Serous	3 (following subsequent surgery)	Isolated (14)
6	51	Serous	1	Bone metastatic (15)
7	53	Serous	4	Solitary (15)
8	59	Serous	9	Solitary (16)
9	45	Serous	6 (following subsequent surgery)	Solitary (17)
10	43	Angiosarcoma	2	Solitary (18)
11	59	Serous	6	Solitary (19)
12	72	Carcinosarcoma	4	Solitary (20)
13	57	Serous	12	Solitary (21)
14	55	Serous	2	Solitary
15	46	Serous	1	Disseminated in the omentum majus and pelvic cavity
16	80	Serous	8	Solitary
17	57	Serous	2	Solitary
18	59	Serous	1	Solitary
19	49	Serous	11	Solitary
20	52	Serous	3	Solitary
21	66	Endometrial adenocarcinoma	3	Disseminated in the omentum majus and the abdominal cavity
22	70	Serous	1	Solitary, sporadic
23	53	Serous	3	a
24	45	Serous	3	a
25	55	Serous	Simultaneity	a
26	43	Serous	4	a
27	64	Serous	Simultaneity	a
28	47	Serous	Simultaneity	a
29	53	Serous	2	a
30	55	Serous	Simultaneity	a
31	45	Serous	2	a
32	59	Serous	2	a
33	61	Serous	4	a
34	48	Serous	5	a

a Information not provided; 1–13, data from case reports published in Western countries (references may be found on Pubmed); 14–34, data from case reports published in China (refer to the footnotes which cannot be found in Pubmed).
